# Nets, spray or both? The effectiveness of insecticide-treated nets and indoor residual spraying in reducing malaria morbidity and child mortality in sub-Saharan Africa

**DOI:** 10.1186/1475-2875-12-62

**Published:** 2013-02-13

**Authors:** Nancy Fullman, Roy Burstein, Stephen S Lim, Carol Medlin, Emmanuela Gakidou

**Affiliations:** 1 Institute for Health Metrics and Evaluation, University of Washington, Seattle, WA, USA; 2 Independent Consultant, Seattle, WA, USA

**Keywords:** Malaria, Insecticide treated nets, Indoor residual spraying, Effectiveness

## Abstract

**Background:**

Malaria control programmes currently face the challenge of maintaining, as well as accelerating, the progress made against malaria with fewer resources and uncertain funding. There is a critical need to determine what combination of malaria interventions confers the greatest protection against malaria morbidity and child mortality under routine conditions.

**Methods:**

This study assesses intervention effectiveness experienced by children under the age of five exposed to both insecticide-treated nets (ITNs) and indoor residual spraying (IRS), as compared to each intervention alone, based on nationally representative survey data collected from 17 countries in sub-Saharan Africa.

**Results:**

Living in households with both ITNs and IRS was associated with a significant risk reduction against parasitaemia in medium and high transmission areas, 53% (95% CI 37% to 67%) and 31% (95% CI 11% to 47%) respectively. For medium transmission areas, an additional 36% (95% CI 7% to 53%) protection was garnered by having both interventions compared with exposure to only ITNs or only IRS. Having both ITNs and IRS was not significantly more protective against parasitaemia than either intervention alone in low and high malaria transmission areas. In rural and urban areas, exposure to both interventions provided significant protection against parasitaemia, 57% (95% CI 48% to 65%) and 39% (95% CI 10% to 61%) respectively; however, this effect was not significantly greater than having a singular intervention. Statistically, risk for all-cause child mortality was not significantly reduced by having both ITNs and IRS, and no additional protectiveness was detected for having dual intervention coverage over a singular intervention.

**Conclusions:**

These findings suggest that greater reductions in malaria morbidity and health gains for children may be achieved with ITNs and IRS combined beyond the protection offered by IRS or ITNs alone.

## Background

Notable strides have been made in reducing the global malaria burden, but the disease remains a substantial source of illness and mortality [1-3]. Insecticide-treated nets (ITNs) and indoor residual spraying (IRS) are two vector control measures currently used in the prevention of malaria transmission. In several countries in sub-Saharan Africa, household ownership of ITNs has been scaled up rapidly over the last few years [1,3,4]. IRS, while typically used for low malaria risk or epidemic-prone regions [5], has been further advocated for use in high and medium malaria transmission settings [6-8]. Numerous studies have investigated the effectiveness of ITNs or IRS separately, but none has conclusively determined whether having both ITNs and IRS provide additional protective benefits, nor the magnitude of additional effectiveness conferred [9,10]. As countries seek to further expand their malaria control programmes and several embark on the next stage of achieving malaria elimination [1,11,12], there is a great need to better understand what combination of interventions are most effective under routine conditions.

Beyond mathematical modelling of ITNs and IRS on health outcomes [13-15], few studies have empirically measured the combined effectiveness of the two interventions. In rural Gambia, a community-based trial is currently exploring the effectiveness of having ITNs and IRS against clinical malaria [16]. In Kenya, Hamel and colleagues showed that household members who were exposed to both ITNs and IRS had significantly greater protection against malaria infection than those who only used ITNs [17]; however, the study did not include a comparison group of IRS-only users. In southern Benin, on the other hand, no significant protective benefits against malaria parasite density were provided by ITNs and IRS combined, as compared to ITNs only [10]. Kleinschmidt and colleagues found that the combination of ITNs and IRS had a larger effect than IRS alone in Zambezia and Bioko, Mozambique [18], but they failed to document a significant interaction between the effects of IRS and ITNs. While these studies suggest that having a combination of ITNs and IRS in households provides greater protection against negative health outcomes, due to study limitations, researchers have been hesitant to strongly and broadly endorse the use of both ITNs and IRS.

In the present study, all available household surveys from sub-Saharan Africa were used. Households were categorized as having ITN only, IRS only, and both ITN and IRS, and the associations between each intervention group and malaria parasite prevalence and all-cause mortality in children under the age of five were assessed. These effects across different malaria transmission areas and urbanicity were also estimated.

## Methods

### Data sources

Analyses included all available Demographic and Health Survey (DHS) and Malaria Indicator Survey (MIS) micro-data for which information about health outcomes, interventions, intervention timing and corresponding covariates were available. Additional file 1 shows the definitions used for each intervention group.

Surveys were excluded if the measured child health outcome (a positive result for parasitaemia or a child death) was not present in a given intervention group. Additional files 2 and 3 present more information on the data sources used.

Five MIS used for the parasitaemia analysis were excluded from mortality analyses because deaths were not recorded (Liberia 2011, Madagascar 2011, Zambia 2006, Zambia 2008 and Zambia 2010). Eight surveys only provided data for whether IRS took place during the previous 12 months rather than the number of months since spraying occurred (Burundi 2010–2011, Burkina Faso 2010–2011, Rwanda 2007–2008, Senegal 2010–2011, Uganda 2011, Zambia 2007, Zimbabwe 2005–2006 and Zimbabwe 2010–2011); however, a proxy IRS exposure metric was computed for these surveys by estimating the average month for the given survey’s national spraying season. This approach was considered an acceptable strategy for these surveys because their national spraying seasons occurred within a two- to three-month span and it is unlikely IRS was received outside spraying campaigns [19-28].

### Ownership of nets

To be classified as a household owning an ITN, nets had to be either: (1) a traditional ITN, which is treated with an insecticide designed to last up to one year and then needs retreatment at least every year thereafter to remain effective; or (2) a long-lasting insecticide-treated net (LLIN), which is impregnated with a type of insecticide meant to be effective for three to five years [3,4]. However, because the DHS and MIS lump all LLINs received over three years ago as “more than 36 months [old]” rather than offering a more precise age measurement, LLINs obtained more than three years ago were excluded. Studies of LLIN insecticide durability under routine conditions suggest that a three-year age limit for LLINs may minimize insecticide integrity issues associated with older mosquito nets [29-31].

Data collection of net characteristics was largely consistent across surveys; the majority of survey interviewers visually validated nets in households and documented: (1) how many months ago each net was obtained; (2) net brand, designating each net as either an LLIN or traditional ITN; and (3) if applicable, how many months ago each net was retreated. Information on whether nets were hanging over sleeping areas was not consistently collected across surveys, so the designation of net ownership relied on the combination of visually confirmed and/or reported nets.

### Indoor residual spraying

Household spraying was ascertained from a combination of two variables in the DHS and MIS: (1) whether the house had ever been sprayed or sprayed within the previous 12 months; and (2) how many months ago spraying took place. For child mortality, surveys which lacked the latter time component for IRS used national reports of the given country and year’s spraying season for information about IRS timing. Twelve months served as the duration threshold for adequate IRS protection, with the exception of one survey (Senegal 2010–2011). For Senegal 2010–2011, reports indicate that the country’s national malaria control programme used a different insecticide solution for the 2010 spraying season, which resulted in a shorter duration of protectiveness provided by IRS nationwide (i e, six months rather the usual twelve) [22]. Subsequently, a six-month duration threshold for IRS protection was applied for the Senegal 2010–2011 survey.

### Health outcomes

For surveys with parasitaemia measurement, the presence of malaria parasites was determined by a rapid diagnostic test (RDT) and/or slide analysis of thick or thin blood smears. While most surveys utilized both types of tests, micro-data and survey reports did not always distinguish whether a positive test result was derived from an RDT or microscopy (Additional file 2).

Child survival from ages one month to 59 months was established by complete birth histories as reported by women of reproductive age (15 to 49 years).

### Malaria transmission risk and seasonality

As described in related work [32], households were assigned to three categories of malaria transmission risk according to their surveys’ primary sampling units (PSUs) and corresponding global positioning system (GPS) coordinates. These levels of malaria transmission risk were classified as: (1) high transmission, with a parasite rate in units of *Pf*PR_2-10_ between 40% and 100%; (2) medium transmission, with a *Pf*PR_2-10_ between 5% and 40%; and (3) low transmission, as with a *Pf*PR_2-10_ between 0% and 5% [33,34]. For surveys completed prior to 2009, *Pf*PR_2-10_ data based on analyses through 2007 were used [35]; for surveys finished between 2009 and 2011, updated *Pf*PR_2-10_ data based on analyses through 2010 were used [36]. The same transmission categories were applied for both 2007 and 2010 *Pf*PR_2-10_ continuous data, but utilizing more recent *Pf*PR_2-10_ data for correspondingly recent surveys was considered methodologically optimal given the rapidly changing malaria epidemiological trends documented in several of countries included in the present study [36]. With a similar extraction technique detailed elsewhere [32], PSU-level seasonality data were applied to households located within survey PSUs.

Studies suggest that the effectiveness of ITNs and IRS may vary by malaria transmission risk [34,37]. In order to examine this effect, individual observations were pooled across individual surveys and categorized observations by malaria transmission risk (high, medium, and low). Each transmission classification was analysed separately for intervention exposure, which was designated by the following four categories: (1) neither intervention; (2) ITN only; (3) IRS only; or (4) both interventions (ITNs and IRS). Having neither ITNs nor IRS served as the reference category against which intervention exposure was compared.

### Effect of net ownership and spraying on parasitaemia by malaria transmission risk

The effect of ITN ownership and/or household spraying on parasitaemia prevalence was measured with a two-step analysis technique [32]: (1) exact matching as a data pre-processing procedure; and (2) logistic regression on the matched dataset.

For each malaria transmission category, children residing in households with at least one intervention (either ITNs or IRS) were matched to children from the same survey without either intervention on the following covariates: (1) child’s age group (one-11 months; 12 to 23 months; 24 to 35 months; 36 to 47 months; and ≥48 months); (2) maternal educational attainment (none, primary or more); (3) urban or rural location of the child’s residence. Exact matching processes were executed with Stata 12’s Coarsened Exact Matching CEM prior to running the logistic regression on the matched dataset [38].

Using the matched datasets for malaria transmission risk, a logistic regression was conducted to control for additional confounding, and then calculated an odds ratio associated with each intervention category. An interaction term was included (ITN*IRS) to explore whether having both interventions was significantly more protective than each intervention on its own. The following covariates were included: (1) child’s age group (one-11 months; 12 to 23 months; 24 to 35 months; 36 to 47 months; and ≥48 months); (2) maternal educational attainment (none, primary, secondary or more); (3) country-specific household wealth quintile; (4) urban or rural location of the child’s residence as designated by each survey; (5) wet or dry season; and (6) survey indicator variables. The weights generated for each set of matched children were included as a sampling weight for the logistic regressions. The same models were applied for urbanicity.

### Effect of net ownership and spraying on child mortality by transmission risk

Complete birth history information from DHS and MIS was used to create retrospective cohorts that track child survival from ages one month to 59 months. Based on household reports about when each net was obtained or retreated from the household net roster and when spraying took place, the household intervention status for ITNs or IRS was ascertained for each month up to three years prior to the survey interview. Previous work on ITNs has extended this cohort back for three years across all surveys [32], but DHS and MIS did not consistently provide IRS information for three years prior to the survey date. Subsequently, cohort length varied by survey, such that Ethiopia 2005 had a three-year retrospective cohort; Namibia 2006–2007 allowed for a two-year cohort; and the other 11 surveys were limited to one year.

With individual surveys pooled by malaria transmission risk, the relationship between interventions and child mortality was assessed with a Cox proportional hazards model. Intervention categories were classified as (1) neither intervention; (2) ITN only; (3) IRS only; and (4) ITNs and IRS, which was computed with the addition of an interaction term (ITNxIRS) to the coefficients generated for ITN only and IRS only. Child age in months served as the analysis time for the hazards model. The following covariates were included for survival analysis: (1) maternal age in 10-year age groups (15–24, 25–34, 35–44, 45+); (2) maternal educational attainment (none, primary, secondary or more); (3) birth interval (less than 24 months, greater than or equal to 24 months or first born); (4) child’s sex; (5) single or multiple birth; (6) household wealth quintile; (7) whether the child’s residence was in an urban location; (8) skilled birth attendance (SBA) coverage at the primary sampling unit (PSU) level; and (9) wet or dry season. A random effect term was also included across surveys in an effort to control for systematic variations across surveys, which was not captured by the covariates. The same models were applied for urbanicity. A pooled model was also run across all observations and found consistent results (data not shown).

All analyses were performed with Stata 12 (Stata Corporation, Texas, USA) and ArcGIS (Ersi, California, USA).

## Results

Additional file 4 provides descriptive statistics for intervention categories and health outcomes by malaria transmission risk. For the parasitaemia analyses, intervention coverage varies substantially by transmission risk: for example, only 2.8% and 9.8% of children who live in high and medium transmission areas respectively have both ITNs and IRS for their homes, whereas 17.7% of children residing in low transmission regions have both interventions. Household application of only IRS is far more common for children residing in low transmission settings (17.7%) than in medium and high transmission areas (7.4% and 0.8%, respectively). Over half of children in high transmission settings resided in households with only ITNs (53.1%), whereas closer to one-third of children experienced only ITNs in both medium (38.9%) and low transmission areas (31.6%). For the mortality analyses, children from medium transmission areas experienced higher coverage of all intervention combinations than their peers in high and low transmission settings.

Additional file 5 details the descriptive statistics for intervention categories and health outcomes by urbanicity. Intervention coverage is similar in urban and rural settings, with the exception of exposure to both ITNs and IRS, which is higher in rural areas (9.1%) than urban areas (4.6%) for the parasitaemia analyses.

### Parasitaemia

**Figure 1:**
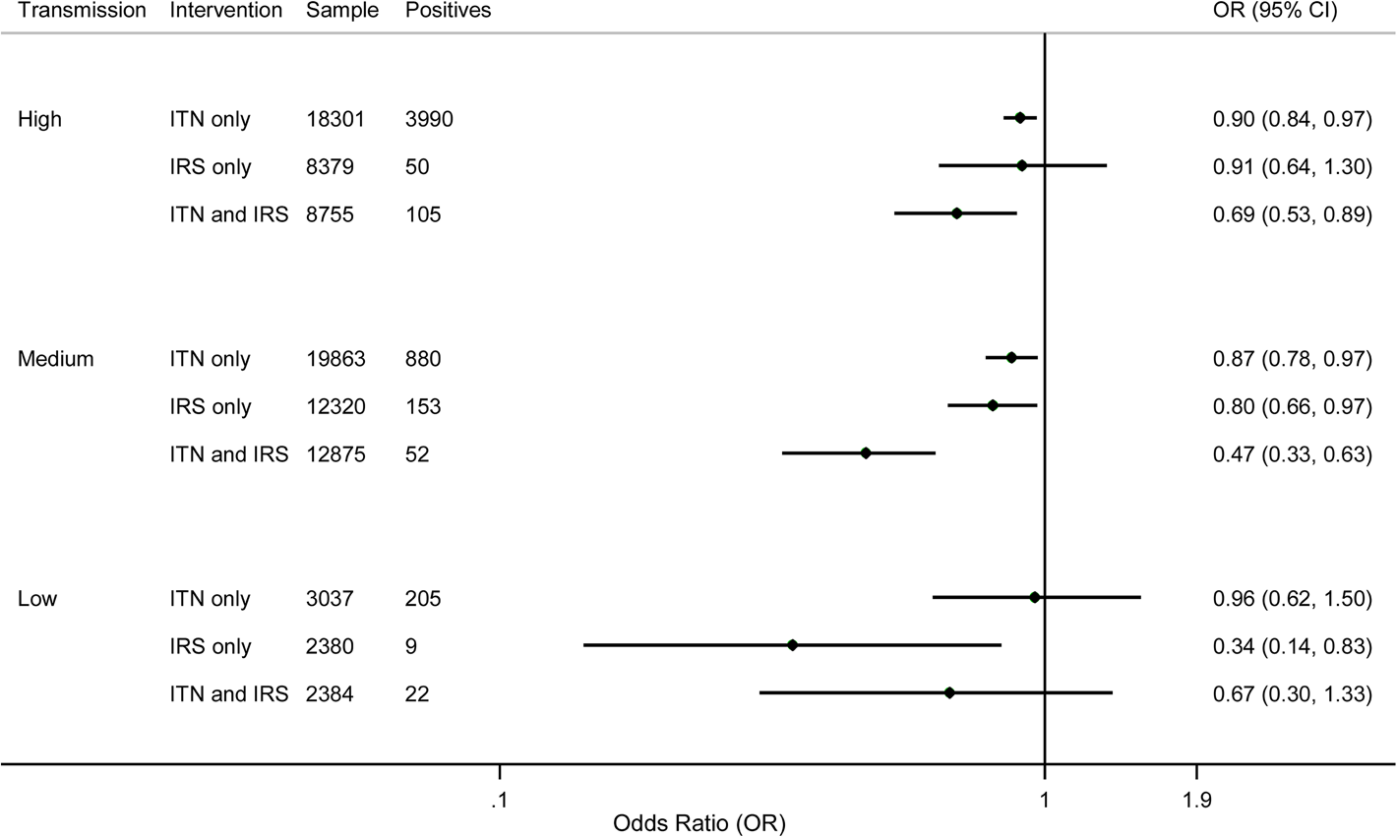
Bivariate effects of ITNs and IRS on parasitaemia prevalence by malaria transmission risk.

Figure 1 shows the results for the association between insecticide-based interventions and parasitaemia by malaria transmission risk. In high malaria transmission areas, having only ITNs was associated with a significant relative risk reduction of 10% (95% CI 3% to 16%, p = 0.004) while having only IRS was not associated with a statistically significant effect against parasitaemia (9% (95% CI −30% to 36%, p > 0.05)). Having both interventions was associated with a relative risk reduction of 31% (95% CI 11% to 47%, p = 0.003); however, the combined effect was not significantly greater than the addition of the individual effects of IRS and ITNs as measured by the interaction term (17% (95% CI −29% to 66%, p > 0.05)). In medium malaria transmission areas, the relative risk reduction in parasitaemia associated with having only ITNs and only IRS was 13% (95% CI 3% to 22%, p = 0.013) and 20% (95% CI 3% to 34%, p = 0.022) respectively. A 53% (95% CI 37% to 67%, p <0.0001) relative risk reduction in parasitaemia was associated with having both ITNs and IRS in medium malaria transmission areas; this effect was significantly greater than the addition of the individual effects (i e, an additional relative risk reduction of 34% (95% CI 7% to 53%, p < 0.018). In low malaria transmission settings, children in households with only IRS experienced a significant risk reduction for parasitaemia of 66% (95% CI 17% to 86%, p = 0.018); however, due to the small number of cases, the uncertainty around this estimate is very large. The other two intervention categories were not associated with significant reductions in parasitaemia. Additional file 6 details results for interventions and parasitaemia by malaria transmission risk. Overall, wet season and increased age were associated with increased odds for parasitaemia, while greater maternal education, greater household wealth, and living in an urban area were all associated with lower odds of parasitaemia.

**Figure 2:**
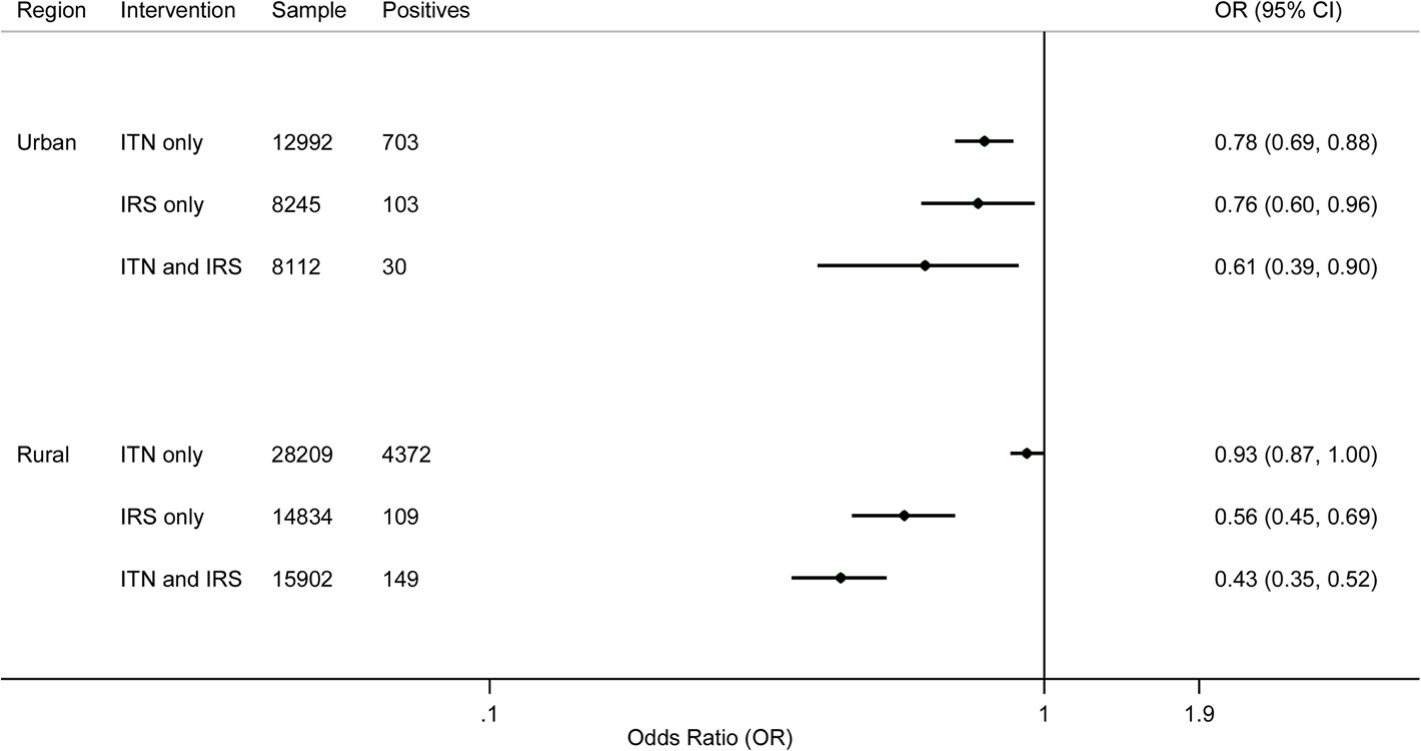
Bivariate effects of ITNs and IRS on parasitaemia prevalence by urbanicity.

Figure 2 shows the results for the association between insecticide-based interventions and parasitaemia by urbanicity. In rural areas, having only ITNs and only IRS were associated with a significant risk reduction in parasitaemia, 7% (95% CI 0% to 13%, p = 0.044) and 44% (95% CI 10% to 42%, p < 0.0001). Having both ITNs and IRS provided a 57% (95% CI 48% to 65%, p < 0.0001) risk reduction in parasitaemia; however, this was not significantly greater than the addition of the individual effects of IRS and ITNs (18% (95% CI −7% to 36%, p > 0.05)). In urban areas, having only ITNs and only IRS were associated with a reduced risk for parasitaemia, 22% (95% CI 12% to 31%, p < 0.0001) and 24% (95% CI 4% to 40%, p = 0.021), respectively. Having both ITNs and IRS showed a 39% (95% CI 10% to 61%, p = 0.010) risk reduction in parasitaemia for children living in urban areas. This was not significantly greater than the sum of the individual effects (0% (95% CI −59% to 36%, p > 0.05)). Additional file 7 shows the findings for interventions and parasitaemia by urbanicity of residence. Overall, wet season, increased child age and high malaria transmission risk were associated with increased odds for parasitaemia, while greater maternal education, low malaria transmission risk and greater household wealth were related to having lower odds for parasitaemia.

### Child mortality

**Figure 3:**
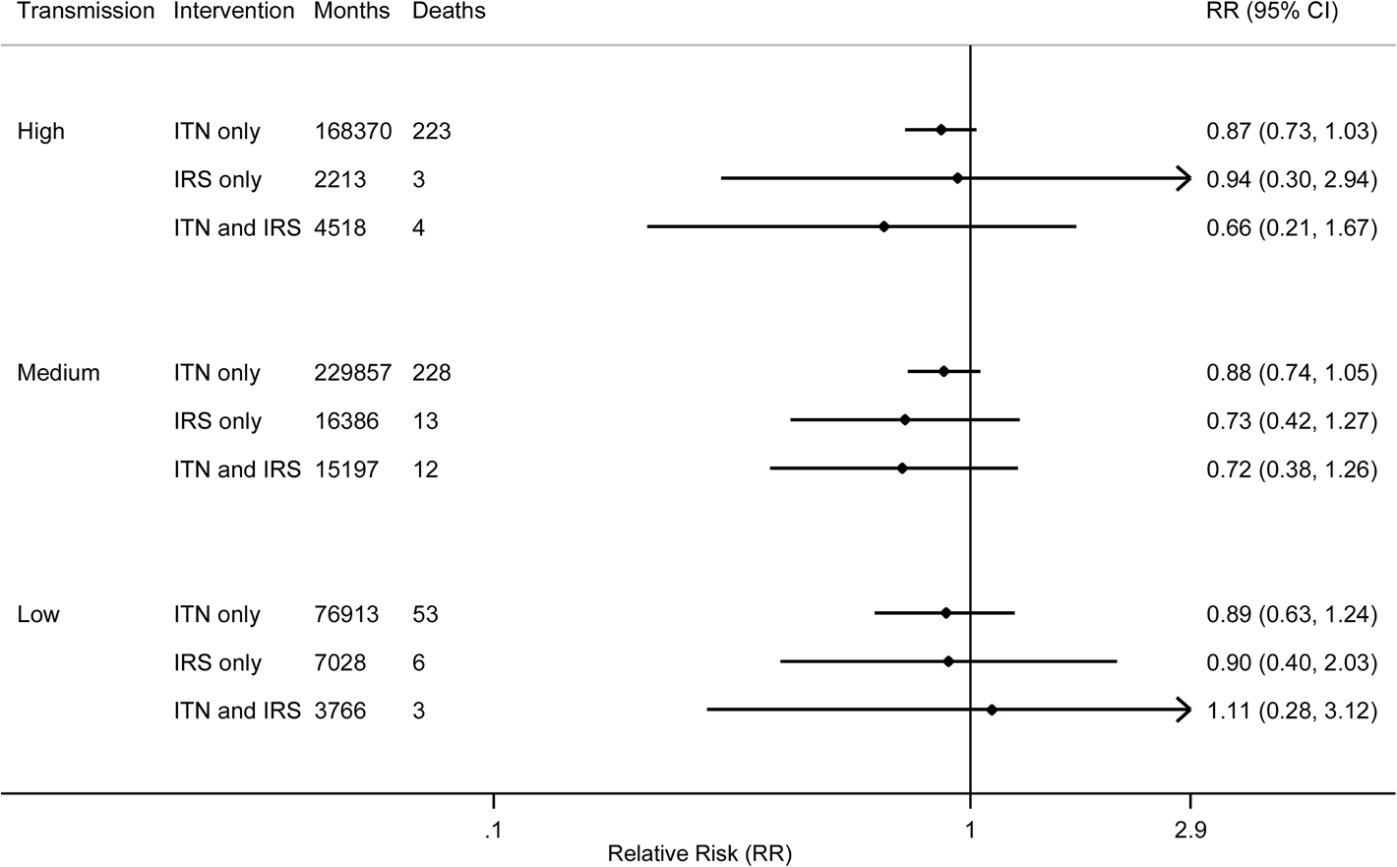
Bivariate effects of ITNs and IRS on child mortality by malaria transmission risk; months refer to observed child-months included in the analysis.

**Figure 4:**
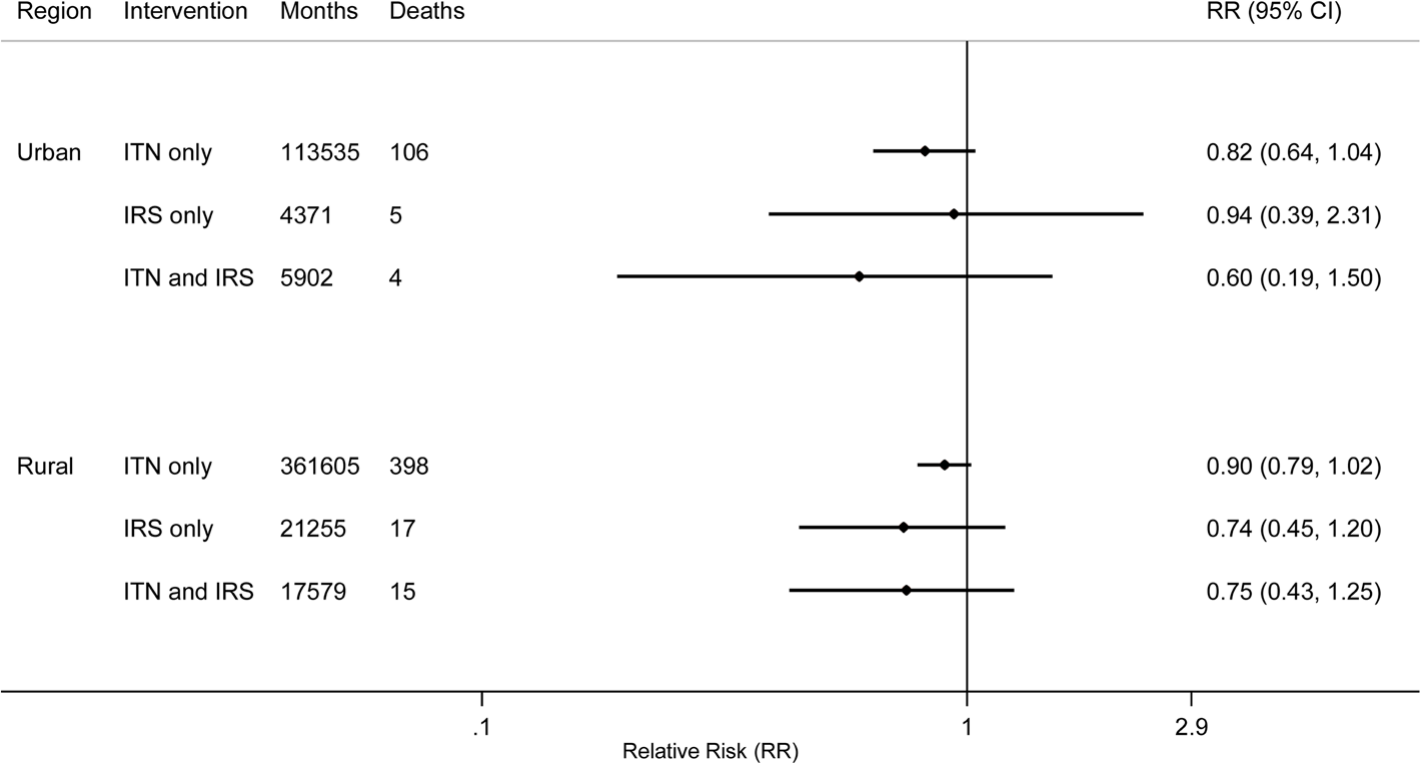
Bivariate effects of ITNs and IRS on child mortality by urbanicity; months refer to observed child-months included in the analysis.

Pooled analyses by transmission and urbanicity were run on the subset of surveys from which PSU-level SBA coverage could be computed; the included surveys were all DHS (*n* = 11) and two MIS (Angola 2006–2007 and Senegal 2008–2009). No intervention or combination of interventions was found to be significantly protective across stratification by transmission or urbanicity category (Figures 3 and 4). The same findings resulted from pooling all observations without transmission or urbanicity stratification (data not shown).

Additional files 8 and 9 detail the results of the logistic regression on under-five mortality. Overall, increased maternal age, multiple births, and birth intervals less than 24 months were all significantly associated with higher under-five mortality, while greater maternal education, higher PSU-level SBA coverage, and larger household sizes were all significantly associated with lower under-five mortality.

## Discussion

This study represents the first multi-country analysis of the combined effectiveness of ITNs and IRS on parasitaemia and under-five mortality under routine conditions. The effect of these interventions was found to vary across malaria transmission levels, such that ITNs are associated with a significant reduction in malaria morbidity in high and medium transmission settings, while IRS appears to be most effective in medium and low transmission areas. The use of both interventions together shows more protection than each intervention on its own; particularly in medium transmission settings, the present study’s results demonstrate a synergistic effect of ITNs and IRS. No statistically significant effects were found for the interventions on child mortality; however, this is likely largely due to the small sample sizes and the very small observed number of deaths in each intervention category.

It is particularly interesting that the risk of parasitaemia was significantly reduced when both interventions were used jointly in areas of medium and high transmission, 53% (95% CI 37% to 67%) and 31% (95% CI 11% to 47%) correspondingly. Further, an additional 34% (95% CI 7% to 53%) effect against parasitaemia was accrued by having both ITNs and IRS compared to the sum of the individual protectiveness provided by ITNs and IRS in medium transmission areas. When the analysis was conducted stratified by location, risk reduction for parasitaemia was also significant for children receiving both interventions in rural and urban areas alike, 57% (95% CI 48% to 65%) and 39% (95% CI 10% to 61%), respectively. The additional protectiveness of having both ITNs and IRS, as compared to each intervention on its own, was not statistically significant in this analysis.

The results for combined intervention approach were less conclusive in low transmission areas. It is likely that the analysis was underpowered to detect significant effects if they existed. For example, under low transmission settings, only 22 children tested positive for malaria parasites for the combined intervention variable. While this finding aligns with this setting's transmission classification (i e, relatively low malaria transmission risk), having so few observations for the health outcome of interest makes detecting a significant effect, if it exists, very difficult. The 95% confidence intervals are quite wide and overlap substantially across intervention categories, further demonstrating the uncertainty in the findings of the present study in low transmission settings. Further, it is possible that other types of interventions are used and that the ways in which ITNs and IRS are deployed in low transmission settings vary (e g, active case detection, focal application of IRS) from the ways that ITNs and IRS are used for areas with greater malaria transmission [39]. Subsequently, the analytical focus of the present study may not have been optimally aligned with the interventions most widely used in areas with low malaria transmission.

For the mortality analyses, it is likely that the small sample sizes of children who had both interventions limit the power of the analysis to detect a significant effect, if one existed. Under high transmission settings, for example, the 95% confidence intervals for relative risk reduction associated with having both interventions (ITNs and IRS) ranged from −67% to 79%, reflecting the very small number of child deaths that occurred during joint intervention exposure (*n =* 4). Similarly, very few child deaths were recorded under IRS only exposure across all malaria risk categories, ranging from three to thirteen fatalities in high and medium transmission areas, respectively. It is thus important for future studies to include additional survey data or consider alternate data sources for analysing the combined effect of ITNs and IRS, as well as the singular effect of IRS, on child mortality.

In many ways, it is not surprising to find relatively small sample sizes for the joint use of ITNs and IRS, regardless of transmission risk or urbanicity. Few malaria control programmes and development agencies in sub-Saharan Africa have actually scaled-up the coverage of both ITNs and IRS. This tendency to invest in one or the other of two interventions, rather than both, likely stems from financial and logistical constraints, as well as the lack of scientific evidence supporting a combined approach [9]. On the other hand, much more data have been collected on the effectiveness of exposure to ITNs and IRS individually. There is substantial evidence both from pooled randomized-control trials (RCTs) [40] and from observational studies that use and ownership of ITNs results in reductions in child mortality and parasitaemia [32,41,42]. Less data are available on the effectiveness of IRS [43]; however, a meta-regression analysis across a range of study types recently showed a 62% (95% CI 54% to 69%) reduction in malaria prevalence [7]. The present study’s findings show that comparable protectiveness against malaria morbidity can be achieved under routine conditions with IRS in areas with low malaria transmission, 66% (95% CI 17% to 86%), as well as in rural areas, 47% (95% CI 31% to 55%). With little existing evidence systematically demonstrating the association between IRS application and mortality reduction [44], further research is needed, especially as more country programmes expand their IRS operations.

The idea that the joint exposure to ITNs and IRS could provide significantly more protection than either intervention alone is not a new one [9], but little was known about the effects of these interventions when used together under routine settings. Until now, no multi-country analysis had previously shown significant results in favour of the combined use of ITNs and IRS against malaria morbidity. The operational and biological mechanisms by which the combined use of ITNs and IRS may provide greater protection than each intervention alone is supported by field studies and modelling exercises. ITNs provide physical protection against mosquitoes and malaria transmission, which is largely conferred to the individual using the net. These nets’ insecticidal properties further deter transmission to the individual, but may also help prevent continued transmission to other household members and nearby community members. IRS is less directly protective of any given individual but garners greater protection to larger groups of people, repelling malaria-transmitting vectors from entering households in the first place or killing the mosquitoes if they rest post-feeding on recently sprayed walls. Subsequently, the combined deployment of ITNs and IRS targets mosquitoes at multiple, complementary transmission points and is likely to most effectively minimize the number of opportunities for malaria vectors to reach any given individual than a singular intervention [9]. Also, when used in combination, the insecticidal protection provided by ITNs and IRS may last longer than when only one insecticide-based intervention is used [44]. Based on results from two areas in Mozambique, which would be classified as medium malaria transmission risk [35], Kleinschmidt and colleagues documented that having both ITNs and IRS provides a multiplicative protective effect against malaria infection beyond the added benefits accrued by having each intervention alone [18]. The present study’s findings support these results and strengthen the evidence base for viewing the protection offered by having both ITNs and IRS as synergistic, especially in settings under which medium malaria transmission are experienced.

In sub-Saharan Africa today, countries face challenging decisions about the financing and prioritization of interventions to prevent malaria, upholding their fragile successes in malaria control, and the possibility of eliminating malaria from their borders. It is not enough to know whether malaria interventions are reaching the populations who need them. Understanding if, and to what extent, these interventions are related to health outcomes under every-day, routine conditions is essential. The present study’s findings offer a critical step toward this understanding, but they must be balanced with timely information on countries’ malaria programme needs and changing epidemiological profiles. The need to further assess the protectiveness of ITNs and IRS in low malaria transmission settings only intensifies, as is understanding how different intervention combinations work over time as countries shift their programmatic strategies along the control to elimination spectrum [39,45]. At a time when donor financial assistance is potentially flat-lining [46] and increasing challenges face the malaria community (e g, the documentation of insecticide resistance in some areas in the world), trying to expand programmes, let alone potentially “doubling-up” the receipt of intervention, involves substantial political will, evidence-based cost-effectiveness analyses, and strategic use of resources.

The present study’s findings need to be interpreted in light of the limitations of the analysis. First, the small sample sizes especially in mortality, limit the power to detect statistical significance. Second, attempts were made to control for as many confounders as were analytically appropriate and plausible; nonetheless, residual confounding may still be present since this is a non-randomized study. Third, the effect of intervention integrity was not investigated as it was associated with health outcomes. For ITNs, data were not available for whether the nets had been washed, which potentially compromises the net’s insecticide potency, or had sizeable holes [47]. For IRS, survey data did not indicate the type of insecticide used for spraying, which is a potentially important factor because the duration of IRS efficacy varies by insecticide applied and countries do not generally use only one type for IRS [31]. Finally, the present study focused on individual exposure to ITNs and IRS and did not further consider community-level effects accrued by either intervention. However, studies show that communal effects for ITNs and IRS may be conferred within smaller ranges than what surveys can capture (e g, neighbouring households within 100 to 300 m, as opposed to the expanse of a whole village, which is represented by the survey PSU) [47-50].

Using publicly available survey data, the present study has sought to quantify the association between having a combination of insecticide-based interventions and child health outcomes. Overall the findings suggest that the combined use of IRS and ITNs provides greater protection against malaria than the use of IRS or ITNs alone. In addition, in medium malaria transmission areas, these results suggest that there may be a synergistic effect of using ITNs and IRS together. While continued work is necessary in order to fully understand how ITNs and IRS are related to child mortality, these findings provide a scientific basis for viewing the combination of ITNs and IRS, under medium malaria transmission settings, as more protective against malaria morbidity than having singular intervention and begin filling the knowledge gap considering the differential effectiveness of ITNs and IRS in sub-Saharan Africa.

## Competing interests

The authors declare that they have no competing interests.

## Authors’ contributions

NF conceived of the study, conducted analyses and drafted the manuscript. RB helped to extract and analyse *Pf*PR data from the Malaria Atlas Project (MAP) group. SSL, CM and EG provided analytical support and manuscript review. All authors read and approved of the final manuscript.

## Supplementary Material

Additional file 1 Classifications of household intervention exposure. Click here for file

Additional file 2 **Surveys included in the analysis for parasitaemia prevalence.** ITN and IRS coverage estimates are at the national level. Percentage of households by season and transmission area is based on the number of households included for each sub-analysis. Click here for file

Additional file 3 **Surveys included in the analysis for child mortality.** ITN and IRS coverage estimates are at the national level. Percentage of households by season and transmission area is based on the number of households included for each sub-analysis. Click here for file

Additional file 4 **Descriptive statistics for intervention coverage and health outcomes by sub-analyses (parasitaemia and child mortality) for malaria transmission risk.** For intervention coverage, units of observation are children under 5 years for parasitaemia and children under 5 years who ever experienced the intervention during analysis exposure time for mortality. Click here for file

Additional file 5 **Descriptive statistics for intervention coverage and health outcomes by sub-analyses (parasitaemia and child mortality) across urbanity.** For intervention coverage, units of observation are children under 5 years for parasitaemia and children under 5 years who ever experienced the intervention during analysis exposure time for mortality. Click here for file

Additional file 6 Logistic regression results for parasitaemia by malaria transmission risk. Click here for file

Additional file 7 Logistic regression results for parasitaemia by urbanicity. Click here for file

Additional file 8 Relative risk results for all-cause child mortality by malaria transmission risk. Click here for file

Additional file 9 Relative risk results for all-cause child mortality by urbanicity. Click here for file
