# Supplementary material for: Nets, spray or both? The effectiveness of insecticide-treated nets and indoor residual spraying in reducing malaria morbidity and child mortality in sub-Saharan Africa
Source: Malar J. 2013 Feb 13;12:62. doi: 10.1186/1475-2875-12-62 (PMC3610288; doi:10.1186/1475-2875-12-62)
Supplement: Additional file 1 — Classifications of household intervention exposure. [file 1475-2875-12-62-S1.pdf]

Additional file 1. Classifications of household intervention exposure.

Intervention Category	Operational Definition
**Control**	Household neither owns 1 ITN nor has the household had its walls sprayed with insecticide in the last 12 months.
**ITN only**	Household owns ≥ 1 ITNs, but the household has not had its walls sprayed with insecticide in the last 12 months.
**IRS only**	Household does not own ≥ 1 ITNs, but the household has had its walls sprayed with insecticide in the last 12 months.
**ITN and IRS**	Household owns ≥ 1 ITNs and has had its walls sprayed with insecticide in the last 12 months.
