# Supplementary material for: Nets, spray or both? The effectiveness of insecticide-treated nets and indoor residual spraying in reducing malaria morbidity and child mortality in sub-Saharan Africa
Source: Malar J. 2013 Feb 13;12:62. doi: 10.1186/1475-2875-12-62 (PMC3610288; doi:10.1186/1475-2875-12-62)
Supplement: Additional file 2 — Surveys included in the analysis for parasitaemia prevalence. ITN and IRS coverage estimates are at the national level. Percentage of households by season and transmission area is based on the number of households included for each sub-analysis. [file 1475-2875-12-62-S2.pdf]

Additional file 2. Surveys included in the analysis for parasitemia prevalence. ITN and IRS coverage estimates are at the national level. Percentage of households by season and transmission area is based on the number of households included for each sub-analysis.

Country	Survey	Year of Survey	No. of Households Sampled	% Households Surveyed by Season		% Households in Transmission Area			% ITN Ownership	% IRS	Parasitemia Measurements		Parasite Test Type	
				Wet	Dry	High	Med.	Low			Total No. Children	No. Positive	RDT	Microscopy
**Angola**	MIS	2006-2007	2,599	7.8	92.2	44.9	53.1	2.0	26.6	3.8	799	174	Yes	Yes*
**Angola**	MIS	2011	7,957	47.9	52.1	20.9	71.9	7.2	32.4	7.0	2,935	292	Yes	Yes
**Burkina Faso**	DHS	2010-2011	14,424	68.6	31.4	93.7	0.0	6.3	56.9	0.9	5,727	3,715	Yes	Yes
**Cameroon**	DHS	2011	14,214	66.2	33.8	75.5	23.5	1.0	36.4	2.3	4,539	1,408	Yes	Yes
**Liberia**	MIS	2011	4,162	100	0.0	51.2	45.2	3.6	47.2	10.7	2,550	843	Yes	Yes
**Madagascar**	MIS	2011	8,094	38.6	61.4	30.5	66.8	2.7	75.6	45.7	5,418	215	Yes	Yes
**Nigeria**	MIS	2010	5,811	79.5	20.5	73.3	26.4	0.3	37.5	0.01	4,606	1,763	Yes	Yes
**Rwanda**	DHS	2007-2008	7,377	7.8	92.2	2.4	52.2	45.4	50.5	6.2	1,478	39	Yes	Yes
**Senegal**	MIS	2008-2009	9,291	17.2	82.8	0.0	97.2	2.8	57.5	10.5	1,526	147	Yes	Yes
**Senegal**	DHS	2010-2011	7,748	69.9	30.1	23.4	71.3	5.3	65.0	4.3	3,348	113	Yes	Yes
**Tanzania**	MIS	2007-2008	8,497	65.0	35.0	21.3	43.5	35.2	44.5	25.1	3,792	477	Yes	n/a
**Uganda**	MIS	2009-2010	4,421	86.1	13.9	62.7	35.6	1.7	42.5	3.5	1,099	458	Yes**	Yes**
**Zambia**	MIS	2006	2,967	11.1	88.9	0.0	85.7	14.3	38.8	9.5	630	77	Yes***	Yes***
**Zambia**	MIS	2008	4,405	24.9	75.1	0.0	90.0	10.0	62.4	14.9	1,497	221	Yes***	Yes***
**Zambia**	MIS	2010	4,361	24.3	75.7	5.2	83.8	11.2	55.2	23.1	1,495	347	Yes***	Yes***

Notes: **Microscopy was used for a sub-population of the survey; **Children were supposed to be diagnosed with both RDT and microscopy, but a small percentage was diagnosed with only the RDT or microscopy; ***Both RDTs and microscopy were supposed to be used for diagnosis, but no data were available differentiating diagnosis type.*
