# Supplementary material for: Nets, spray or both? The effectiveness of insecticide-treated nets and indoor residual spraying in reducing malaria morbidity and child mortality in sub-Saharan Africa
Source: Malar J. 2013 Feb 13;12:62. doi: 10.1186/1475-2875-12-62 (PMC3610288; doi:10.1186/1475-2875-12-62)
Supplement: Additional file 3 — Surveys included in the analysis for child mortality. ITN and IRS coverage estimates are at the national level. Percentage of households by season and transmission area is based on the number of households included for each sub-analysis. [file 1475-2875-12-62-S3.pdf]

Additional file 3. Surveys included in the analysis for child mortality. ITN and IRS coverage estimates are at the national level. Percentage of households by season and transmission area is based on the number of households included for each sub-analysis.

Country	Survey	Year of Survey	No. of Households Sampled	% Households Surveyed by Season		% Households in Transmission Area			% ITN Ownership	% IRS	Survival Analysis	
				Wet	Dry	High	Med.	Low			Total No. Children	No. Child Deaths
**Angola**	MIS	2006-2007	2,599	8.5	91.6	45.5	52.6	1.9	26.6	3.8	2,560	34
**Burundi**	DHS	2010-2011	8,596	31.3	68.7	1.1	75.1	23.8	52.0	0.3	8,125	88
**Burkina Faso**	DHS	2010-2011	14,424	68.7	31.3	93.8	0.0	6.2	56.9	0.9	15,237	220
**Cameroon**	DHS	2011	14,214	65.5	35.5	74.7	24.2	1.1	36.4	2.3	11,286	182
**Ethiopia**	DHS	2005	13,721	25.1	74.9	0.0	44.6	55.4	3.5	8.4	13,739	363
**Kenya**	DHS	2008-2009	9,057	38.4	61.6	3.2	47.3	49.5	48.9	6.9	6,142	50
**Malawi**	DHS	2009-2010	24,825	0.6	99.4	56.4	43.6	0.0	55.0	2.8	20,686	277
**Namibia**	DHS	2006-2007	9,200	44.8	55.2	0.0	59.0	41.0	15.6	20.4	5,009	93
**Rwanda**	DHS	2007-2008	7,377	7.8	92.2	3.2	52.5	44.3	50.5	6.2	5,626	42
**Senegal**	MIS	2008-2009	9,291	18.1	81.9	0.0	96.5	3.5	57.5	10.5	15,653	141
**Senegal**	DHS	2010-2011	7,748	7.8	92.2	2.0	64.9	33.1	65.0	4.3	12,260	80
**Uganda**	DHS	2006	8,870	82.4	17.6	35.5	59.7	4.8	19.1	6.7	7,319	122
**Uganda**	DHS	2011	9,033	82.3	17.7	55.1	40.1	4.8	59.8	7.2	7,793	77
**Zambia**	DHS	2007	7,164	6.2	93.8	0.2	99.8	0.0	54.2	15.6	6,248	84
**Zimbabwe**	DHS	2005-2006	9,285	14.7	85.3	0.0	24.4	75.6	6.9	15.1	5,224	56
**Zimbabwe**	DHS	2010-2011	9,465	21.9	78.1	0.4	43.8	55.8	25.8	18.8	5,278	44
