# Supplementary material for: Nets, spray or both? The effectiveness of insecticide-treated nets and indoor residual spraying in reducing malaria morbidity and child mortality in sub-Saharan Africa
Source: Malar J. 2013 Feb 13;12:62. doi: 10.1186/1475-2875-12-62 (PMC3610288; doi:10.1186/1475-2875-12-62)
Supplement: Additional file 4 — Descriptive statistics for intervention coverage and health outcomes by sub-analyses (parasitaemia and child mortality) for malaria transmission risk. For intervention coverage, units of observation are children under 5 years for parasitaemia and children under 5 years who ever experienced the intervention during analysis exposure time for mortality. [file 1475-2875-12-62-S4.pdf]

Additional file 4. Descriptive statistics for intervention coverage and health outcomes by sub-analyses (parasitemia and child mortality) for malaria transmission risk. For intervention coverage, units of observation are children under 5 years for parasitemia and children under 5 years who ever experienced the intervention during analysis exposure time for mortality.

			**% Intervention Coverage**			**Health Outcomes**	
**Analysis**	**Level**		**ITN only**	**IRS only**	**ITN and IRS**	**Para. Prevalence (%)**	**No. Deaths**
**Parasitemia**	Transmission	High	53.1	0.8	2.8	40.4	-
		Medium	38.9	7.4	9.8	11.7	-
		Low	31.6	17.7	17.7	7.3	-
**Child Mortality**	Transmission	High	50.5	2.4	1.8	-	590
		Medium	50.7	9.2	5.4	-	780
		Low	31.9	6.0	2.6	-	382
