# Supplementary material for: Nets, spray or both? The effectiveness of insecticide-treated nets and indoor residual spraying in reducing malaria morbidity and child mortality in sub-Saharan Africa
Source: Malar J. 2013 Feb 13;12:62. doi: 10.1186/1475-2875-12-62 (PMC3610288; doi:10.1186/1475-2875-12-62)
Supplement: Additional file 5 — Descriptive statistics for intervention coverage and health outcomes by sub-analyses (parasitaemia and child mortality) across urbanity. For intervention coverage, units of observation are children under 5 years for parasitaemia and children under 5 years who ever experienced the intervention during analysis exposure time for mortality. [file 1475-2875-12-62-S5.pdf]

Additional file 5. Descriptive statistics for intervention coverage and health outcomes by sub-analyses (parasitemia and child mortality) across urbanity. For intervention coverage, units of observation are children under 5 years for parasitemia and children under 5 years who ever experienced the intervention during analysis exposure time for mortality.

			% Intervention Coverage			Health Outcomes	
**Analysis**	**Level**		**ITN only**	**IRS only**	**ITN and IRS**	**Para. Prevalence (%)**	**No. Deaths**
**Parasitemia**	Urbanicity	Rural	46.2	5.9	9.1	25.8	-
		Urban	38.4	5.6	4.6	15.5	-
**Child Mortality**	Urbanicity	Rural	45.6	6.2	3.4	-	1,402
		Urban	48.6	6.7	4.4	-	350
