# Supplementary material for: Nets, spray or both? The effectiveness of insecticide-treated nets and indoor residual spraying in reducing malaria morbidity and child mortality in sub-Saharan Africa
Source: Malar J. 2013 Feb 13;12:62. doi: 10.1186/1475-2875-12-62 (PMC3610288; doi:10.1186/1475-2875-12-62)
Supplement: Additional file 6 — Logistic regression results for parasitaemia by malaria transmission risk. [file 1475-2875-12-62-S6.pdf]

Additional file 6. Logistic regression results for parasitaemia by malaria transmission risk.

		High Transmission			Medium Transmission			Low Transmission		
**Covariate**		OR	*p*	95% CI	OR	*p*	95% CI	OR	*p*	95% CI
ITN only		0.90	0.004	(0.84, 0.97)	0.87	0.013	(0.78, 0.97)	0.96	0.883	(0.62, 1.50)
IRS only		0.91	0.604	(0.64, 1.30)	0.80	0.022	(0.66, 0.97)	0.34	0.018	(0.14, 0.83)
ITN and IRS		0.69	0.003	(0.53, 0.89)	0.47	0.000	(0.33, 0.63)	0.67	0.205	(0.30, 1.33)
Seasonality	Dry	1.00	-	-	1.00	-	-	1.00	-	-
	Wet	1.52	0.000	(1.40, 1.66)	1.93	0.000	(1.74, 2.15)	3.46	0.002	(1.57, 7.64)
Child's age (in months)	1 to 11	1.00	-	-	1.00	-	-	1.00	-	-
	12 to 23	1.35	0.000	(1.20, 1.52)	1.63	0.000	(1.36, 1.95)	1.12	0.705	(0.62, 2.04)
	24 to 35	1.92	0.000	(1.71, 2.16)	2.37	0.000	(1.98, 2.84)	1.94	0.026	(1.08, 3.49)
	36 to 47	2.15	0.000	(1.91, 2.43)	2.74	0.000	(2.29, 3.27)	2.06	0.011	(1.18, 3.57)
	48 to 59	2.42	0.000	(2.15, 2.74)	3.06	0.000	(2.56, 3.66)	1.65	0.091	(0.82, 2.95)
Maternal Education	None	1.00	-	-	1.00	-	-	1.00	-	-
	Primary	0.78	0.000	(0.71, 0.84)	0.98	0.758	(0.88, 1.10)	1.22	0.393	(0.77, 1.94)
	≥ Secondary	0.64	0.000	(0.57, 0.72)	0.69	0.000	(0.58, 0.81)	0.56	0.134	(0.26, 1.20)
Household wealth	Poorest	1.00	-	-	1.00	-	-	1.00	-	-
	Quintile 2	1.02	0.748	(0.92, 1.12)	0.90	0.085	(0.79, 1.02)	0.93	0.754	(0.49, 1.46)
	Quintile 3	0.91	0.055	(0.82, 1.00)	0.87	0.026	(0.76, 0.98)	1.11	0.669	(0.69, 1.79)
	Quintile 4	0.77	0.000	(0.69, 0.86)	0.55	0.000	(0.48, 0.64)	0.62	0.077	(0.36, 1.05)
	Richest	0.48	0.000	(0.42, 0.56)	0.25	0.000	(0.21, 0.31)	0.20	0.000	(0.09, 0.45)
Urban residence	Rural	1.00	-	-	1.00	-	-	1.00	-	-
	Urban	0.45	0.000	(0.42, 0.56)	0.77	0.000	(0.69, 0.85)	0.58	0.020	(0.37, 0.92)
