# Supplementary material for: Nets, spray or both? The effectiveness of insecticide-treated nets and indoor residual spraying in reducing malaria morbidity and child mortality in sub-Saharan Africa
Source: Malar J. 2013 Feb 13;12:62. doi: 10.1186/1475-2875-12-62 (PMC3610288; doi:10.1186/1475-2875-12-62)
Supplement: Additional file 8 — Relative risk results for all-cause child mortality by malaria transmission risk. [file 1475-2875-12-62-S8.pdf]

Additional file 8. Relative risk results for all-cause child mortality by malaria transmission risk.

Covariate		High Transmission			Medium Transmission			Low Transmission		
		RR	*p*	95% CI	RR	*p*	95% CI	RR	*p*	95% CI
ITN only		0.87	0.113	(0.73, 1.03)	0.88	0.158	(0.74, 1.05)	0.89	0.489	(0.63, 1.24)
IRS only		0.94	0.919	(0.30, 2.94)	0.73	0.259	(0.42, 1.27)	0.90	0.800	(0.40, 2.03)
ITN and IRS		0.66	0.659	(0.21, 1.67)	0.72	0.662	(0.38, 1.26)	1.11	0.780	(0.28, 3.12)
Seasonality	Dry	1.00	-	-	1.00	-	-	1.00	-	-
	Wet	1.04	0.685	(0.88, 1.22)	1.10	0.242	(0.94, 1.29)	1.35	0.058	(0.99, 1.84)
Child's sex	Male	1.00	-	-	1.00	-	-	1.00	-	-
	Female	0.91	0.245	(0.77, 1.07)	1.02	0.755	(0.89, 1.18)	0.83	0.064	(0.68, 1.01)
Birth interval	≥ 24 mo.	1.00	-	-	1.00	-	-	1.00	-	-
	< 24 mo.	1.85	0.172	(0.76, 4.47)	3.77	0.000	(2.44, 5.82)	1.38	0.472	(0.57, 3.36)
Birth type	Single	1.00	-	-	1.00	-	-	1.00	-	-
	Multiple	2.65	0.000	(1.96, 3.59)	2.62	0.000	(1.94, 3.54)	2.99	0.000	(1.89, 4.73)
Maternal age (years)	15-24	0.82	0.051	(0.68, 1.00)	0.90	0.210	(0.76, 1.06)	0.82	0.120	(0.63, 1.05)
	25-34	1.00	-	-	1.00	-	-	1.00	-	-
	35-44	1.14	0.234	(0.92, 1.42)	1.42	0.000	(1.18, 1.70)	1.33	0.031	(1.03, 1.73)
	45-49	0.98	0.941	(0.50, 1.90)	0.98	0.956	(0.55, 1.76)	1.58	0.131	(0.87, 2.87)
Maternal Education	None	1.00	-	-	1.00	-	-	1.00	-	-
	Primary	0.92	0.337	(0.77, 1.09)	0.81	0.025	(0.68, 0.97)	0.82	0.180	(0.61, 1.10)
	≥Secondary	0.64	0.006	(0.46, 0.88)	0.59	0.000	(0.45, 0.78)	0.62	0.014	(0.42, 0.91)
No. household members	≤ 4	1.00	-	-	1.00	-	-	1.00	-	-
	5-8	0.62	0.000	(0.51, 0.75)	0.55	0.000	(0.46, 0.65)	0.58	0.000	(0.46, 0.73)
	≥ 9	0.61	0.000	(0.49, 0.76)	0.45	0.000	(0.36, 0.56)	0.37	0.000	(0.26, 0.53)
Household wealth (quintiles)	Poorest	1.00	-	-	1.00	-	-	1.00	-	-
	Quintile 2	0.85	0.157	(0.67, 1.07)	1.10	0.360	(0.90, 1.33)	1.10	0.525	(0.82, 1.47)
	Quintile 3	0.77	0.035	(0.61, 0.98)	0.92	0.429	(0.74, 1.14)	0.96	0.794	(0.71, 1.30)
	Quintile 4	0.86	0.245	(0.67, 1.11)	0.93	0.510	(0.73, 1.17)	0.83	0.295	(0.59, 1.17)
	Richest	0.74	0.078	(0.53, 1.04)	0.66	0.013	(0.47, 0.91)	0.63	0.049	(0.39, 1.00)
Urban residence	Rural	1.00	-	-	1.00	-	-	1.00	-	-
	Urban	0.88	0.354	(0.67, 1.15)	1.63	0.000	(1.31, 2.03)	1.40	0.047	(1.00, 1.94)
PSU-level SBA		0.63	0.003	(0.46, 0.85)	0.75	0.099	(0.53, 1.06)	0.78	0.350	(0.47, 1.31)
